# Oral herpes simplex virus infection in patients undergoing chemotherapy – an integrative review

**DOI:** 10.3205/dgkh000483

**Published:** 2024-05-17

**Authors:** Aristéa Ribeiro Carvalho, Renan Lemos da Silva, Ed Campos Vieira Neto, Mailon Cury Carneiro, Ana Carolina Fragoso Motta, Ana Paula Campanelli, Cassia Maria Fischer Rubira, Paulo Sérgio da Silva Santos

**Affiliations:** 1 Department of Surgery, Stomatology, Pathology and Radiology, Bauru School of Dentistry, University of São Paulo, Bauru, Brazil; 2 Department of Biological Sciences, Bauru School of Dentistry, University of São Paulo, Bauru, Brazil; 3 Department of Surgery, Stomatology, Pathology and Radiology, Bauru School of Dentistry, University of São Paulo; 4 Department of Stomatology, Public Health and Forensic Dentistry, School of Dentistry of Ribeirão Preto, University of São Paulo

**Keywords:** Herpes simplex, virus diseases, mouth, antineoplastic agents

## Abstract

**Aim:**

The purpose of this study is to undertake an integrative literature review in order to determine the prevalence, etiology, and reactivation of oral HSV infection in patients receiving chemotherapy (CT).

**Methods:**

The study was carried out in the PubMed/MEDLINE, Embase, Virtual Health Library, and Scopus databases, using the descriptors “Herpes Simplex”, “Viral Diseases”, “Mouth”, and “Antineoplastic Agents”.

**Results:**

The findings suggest that HSV infection is widespread in this group of patients and can be severe. HSV infection is frequent in CT patients, and treatment should begin as soon as it is feasible, utilizing antivirals to avoid future difficulties, as patients are immunocompromised.

**Conclusion:**

It is critical for health professionals to be fully informed on the dangers and treatment choices available, with the most appropriate therapy for each circumstance. Furthermore, more recent research with acceptable methodological rigor is required to better quantify the prevalence of HSV in these patients.

## Zusammenfassung

**Zielsetzung:**

Um die Prävalenz, Ätiologie und Reaktivierung von oralen HSV-Infektionen bei Patienten, die eine Chemotherapie (CT) erhalten, zu bestimmen, wurde eine integrative Literaturanalyse durchgeführt.

**Methode:**

Die Studie wurde in den Datenbanken PubMed/MEDLINE, Embase, Virtual Health Library und Scopus unter Verwendung der Deskriptoren „Herpes simplex“, „Viruskrankheiten“, „Mund“ und „Antineoplastische Mittel“ durchgeführt.

**Ergebnisse:**

Die Ergebnisse weisen darauf hin, dass eine HSV-Infektion in dieser Patientengruppe weit verbreitet ist und schwerwiegend sein kann. HSV-Infektionen sind bei CT-Patienten häufig, und die Behandlung sollte so rasch wie möglich mit antiviralen Mitteln begonnen werden, um künftige Schwierigkeiten zu vermeiden, da die Patienten immungeschwächt sind.

**Schlussfolgerung:**

Es ist von entscheidender Bedeutung, dass die Angehörigen der Gesundheitsberufe umfassend über die Gefahren und die verfügbaren Behandlungsmöglichkeiten informiert sind und die für die jeweilige Situation am besten geeignete Therapie kennen. Darüber hinaus sind neuere Untersuchungen mit akzeptabler methodischer Strenge erforderlich, um die Prävalenz von HSV bei diesen Patienten besser zu quantifizieren.

## Introduction

Herpes simplex virus (HSV) infections are comorbidities with global prevalence. In 2016, it was estimated that approximately 66% of the population (0–49 years of age) worldwide had HSV type 1 (HSV-1) infection in some region of the body and 13.2% (15–49 years of age) lived with HVS type 2 (HSV-2) as latent carriage [1]. HSV-1 is highly related to oral herpes simplex, commonly producing the classic symptoms of mucocutaneous vesicles that coalesce and rupture, with primarily mouth-to-mouth transmission, as opposed to HSV-2 which is more associated with genital infections [1], [2].

Regardless of viral typing, HSV infection pathogenesis characteristically affects skin and mucosa regions, resulting in replication in epithelial cells of the main site of infection [1]. Subsequently, it affects sensory nerve ganglia, promoting the latent phase of infection, characterized by viral replication in its dormant state, a stage responsible for producing the clinical manifestation of reappearances after the first infection [1], [2], [3]. Several stimuli are responsible for promoting HSV reactivation, such as physical and emotional stress, sleep deprivation, fever, and exposure to ultraviolet rays, hormonal imbalance, and immunosuppression of different origins [4].

Patients undergoing chemotherapy (CT), a treatment for different types of cancer, suffer many side effects, which vary depending on the affected organ, tumor stage, CT drug combination, and dosage. One of the side effects of the medications is immunosuppression, which is a key role in HSV reactivation, making such patients more likely to develop the disease with increased severity and comorbidity [5].

The aim of this study was to conduct an integrative review of the literature, focusing on the prevalence of HSV episodes in patients undergoing CT, promoting a better understanding of the possible relationship between CT immunosuppression and HSV infection in individuals undergoing CT without head and neck radiotherapy, and enabling better predictability and specific care for herpes simplex episodes in the oral cavity.

## Methods

The integrative literature review was performed according to Page et al. [6] and included the following steps: subject identification, literature search, study selection based on pre-established inclusion criteria, data analysis and compilation, and presentation of outcomes.

The guiding question of the review was “What is the prevalence of HSV infection in cancer patients treated by chemotherapy?” based on the PECO strategy (P: cancer patients; E: undergoing chemotherapy; C: comparator (not applicable in this study); O: HSV infection).

The search was conducted in the following databases: PubMed/MEDLINE, Embase (via Elsevier), Biblioteca Virtual em Saúde (BVS) and Scopus. The descriptors “Herpes simplex”, “virus diseases”, “mouth” and “antineoplastic agents” were used, as well as their correlated terms, using the Boolean operators “AND” and “OR”. Studies were included if they were available in full and addressed the topic in question, with no language or publication period restrictions. Studies were excluded if they were review articles, theses, dissertations, editorials, case reports, case series, or involved patients undergoing head and neck radiotherapy.

After searching all databases, the retrieved records were transferred to the EndNote Web^®^ reference manager (Clarivate, London, UK) to identify duplicates. Two independent reviewers (A.R.C. and R.L.S) read the titles and abstracts of each article and excluded studies that did not meet the eligibility criteria. Following this pre-selection, full-text reading was performed to analyze which studies exactly met the established criteria. Any discrepancies were resolved through discussion with a third reviewer (P.S.S.S.).

The study data were extracted by two researchers (A.R.C. and R.L.S), and the studies selected for inclusion were extracted using a standardized table including the following information: authors/year/country of origin, type of study, population, sex, age, objective, methodology, conclusion (Table 1 (Tab. 1)).

To tabulate the data, a database comprising the obtained information was employed and structured in a Microsoft^®^ Excel^®^ spreadsheet for Microsoft 365 MSO (version 2301; Microsoft Corporation, Redmond, WA, USA). The information in the selected papers was gathered using a descriptive analysis.

## Results

**Figure 1:**
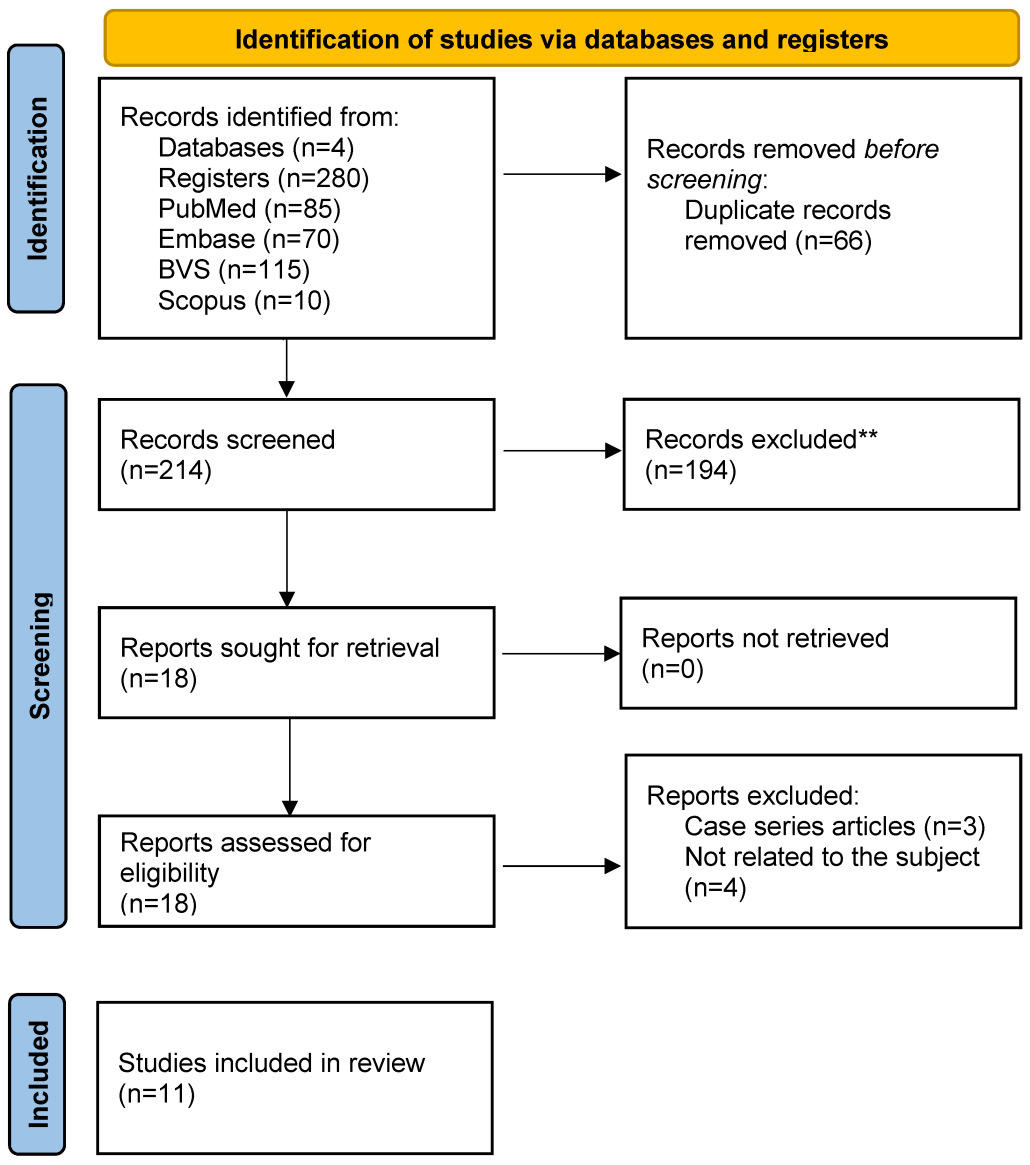
Description of the article search for this integrative review

By searching the different databases and crossing the descriptors, 280 articles were found; 85 in PubMed, 10 in Scopus, 115 in VHL, and 70 in Embase. A total of 66 duplicate articles were removed. The titles and abstracts of 214 articles were then read, after which 18 articles were selected to be read in full. At the end, according to the inclusion and exclusion criteria, 11 articles were selected to compose this review (Figure 1 (Fig. 1))

The findings of this study reflect a wide age range of the individuals studied, ranging from young children aged 6 months to adults aged 84 years. In total, 2,225 patients were analyzed in the 11 papers that comprised this evaluation (Table 1 (Tab. 1)). 

In addition to viral infections, bacterial and fungal infections with the presence of one or more agents were found. In one of the studies, a higher percentage of HSV-associated lesions was observed in patients concomitantly undergoing CT and bone-marrow transplant, especially autologous transplants [7]. Bergmann et al. [8] observed most HSV-associated ulcerated lesions in the alveolar process of patients undergoing CT. Another study reported high prevalence of HSV-1 in children with CT-induced oral mucositis [9]. However, there is disagreement regarding the association of HSV-1 with mucositis severity or age. While some studies state that there is no relationship, others suggest that oral HSV is associated with prolonged mucositis and worse response to initial antibiotic treatment [10]. Although some studies found the classic form of herpes labialis, in most cases, ulcers were seen with association with HSV. Besides oral HSV, other oral manifestations were found in cancer patients undergoing CT, such as gingivitis, caries, mucositis, periodontitis, cheilitis, primary herpetic gingivostomatitis, dry lips, mucosal pallor, mucosal petechiae, and ecchymoses.

Several laboratory techniques were utilized to detect HSV in the studies reviewed here, including polymerase chain reaction (PCR), serology, immunoenzymatic assay, and immunofluorescence from cell culture, swab and blood tests. Aggarwal et al. [9] used PCR (qualitative and quantitative) and ELISA (IgG and IgM) tests and observed that real-time quantitative PCR may be more sensitive in detecting HSV-1 in tissue samples. In 2007, Anirudhan et al. [11] attributed the low incidence of HSV-1 detection to the technique used for laboratory diagnosis (culture and serology), and states that the Tzanck technique could have identified more cases of HSV. This test is performed by scraping the base and sides of a vesicle with a scalpel; the material thus obtained is then stained with Wright’s or Giemsa's stain, which visualizes specific characteristics of HSV such as multinucleated giant cells. These are a sign of infection with herpes simplex or shingles [12].

A decrease in cases of oral mucositis and candidiasis was observed between the years 1997and 2007, but the incidence of HSV cases remained the same. Furthermore, it was concluded that the development of prophylactic protocols is necessary to prevent patients undergoing CT from developing oral lesions [13]. In another study, a comparison was made between groups of HSV-seropositive patients, where one group received prophylactic acyclovir and the other group received placebo. A statistically significant reduction in the incidence of labial herpes was found in the group receiving acyclovir prophylaxis compared to the group receiving placebo [8]. Only one study has reported the treatment used when herpes simplex occurs in cancer patients undergoing CT, using intravenous acyclovir [7].

## Discussion

In the current review, the studies pointed out an association between infections by different microorganisms, highlighting the importance of adequate monitoring of oral health during CT treatment. We observed that the use of a prophylaxis protocol in oncology patients seropositive for HSV-1 undergoing CT may be well indicated, considering the incidence of cases of reactivation due to immunosuppression caused by CT. Moreover, divergent diagnostic laboratory results were observed according to the technique used, showing a need to increase the knowledge of health professionals and employ techniques best suited to these cases, to ensure the most efficacious treatment of lesions.

## Conclusions

There are few studies in the literature that address the prevalence of HSV infection in patients undergoing CT, despite the fact that they are immunocompromised patients who are susceptible to opportunistic infections (either new or reactivations). HSV is the pathogen most commonly associated with viral infections in immunocompromised patients undergoing CT. More studies with better methodological rigor as well as more current investigations are needed to measure the prevalence of HSV infection in oncology patients undergoing CT.

### Limitations

The limitations of the study are that the studies addressing herpes in patients undergoing CT are not standardized. For instance, viral detection methods and information on episodes of herpes simplex prior to CT vary from study to study, thus preventing comparability. In addition, only one study included the treatment regimen used against HSV, and even then, it did not specify doses used, only the mode of administration. A small number of studies have reported the characteristics of the clinical manifestations of oral herpes simplex in the group of patients under CT.

## Notes

### Competing interests

The authors declare that they have no competing interests.

### Funding

This study was financed in part by the Coordenação de Aperfeiçoamento de Pessoal de Nível Superior (CAPES), Brazil (Finance Code 001). 

### Authors’ ORCIDs

- Carvalho AR: 0000-0002-4045-0837
- Silva RL: 0000-0001-5837-410X
- Campos Vieira Neto E: 0000-0003-1445-9771
- Carneiro MC: 0000-0003-3952-6002
- Motta ACF: 0000-0002-3887-9239
- Campanelli AP: 0000-0002-0536-5469
- Rubira CMFG: 0000-0003-2119-1144
- Santos PSS: 0000-0002-0674-3759

## Figures and Tables

**Table 1:**
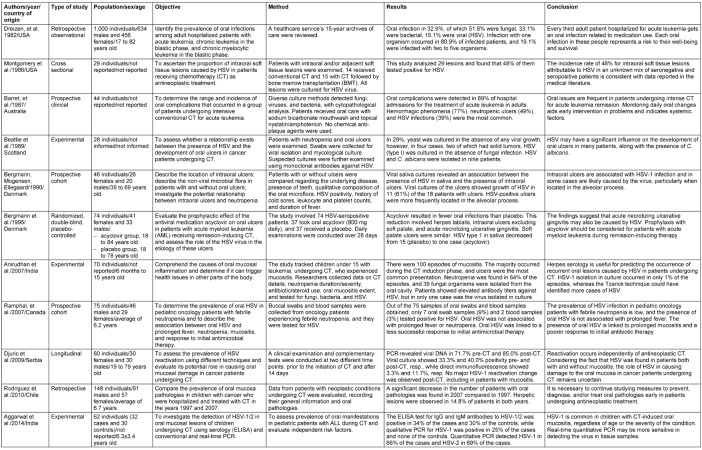
General characteristics of the articles included in this study
